# Spontaneous Clearance of Vertically Acquired Hepatitis C Infection: Implications for Testing and Treatment

**DOI:** 10.1093/cid/ciac255

**Published:** 2022-04-09

**Authors:** A E Ades, Fabiana Gordon, Karen Scott, Intira Jeannie Collins, Claire Thorne, Lucy Pembrey, Elizabeth Chappell, Eugènia Mariné-Barjoan, Karina Butler, Giuseppe Indolfi, Diana M Gibb, Ali Judd

**Affiliations:** Population Health Sciences, University of Bristol Medical School, Bristol, United Kingdom; Population Health Sciences, University of Bristol Medical School, Bristol, United Kingdom; Medical Research Council Clinical Trials Unit, University College London, London, United Kingdom; Medical Research Council Clinical Trials Unit, University College London, London, United Kingdom; Population, Policy and Practice Research and Teaching Department, UCL Great Ormond Street Institute of Child Health, London, United Kingdom; Department of Medical Statistics, London School of Hygiene and Tropical Medicine, London, United Kingdom; Medical Research Council Clinical Trials Unit, University College London, London, United Kingdom; Public Health Department, Université Côte d’Azur, Centre Hospitalier Universitaire de Nice, Nice, France; Children’s Health Ireland at Crumlin and Temple Street, Dublin, Ireland; Meyer Children’s Hospital and Department Neurofarba, University of Florence, Firenze, Italy; Medical Research Council Clinical Trials Unit, University College London, London, United Kingdom; Medical Research Council Clinical Trials Unit, University College London, London, United Kingdom

**Keywords:** hepatitis C virus, HCV, vertical transmission, spontaneous clearance, overtreatment

## Abstract

**Background:**

Current guidelines recommend that infants born to women with hepatitis C virus (HCV) viremia be screened for HCV antibody at age 18 months and, if positive, referred for RNA testing at 3 years to confirm chronic infection. This policy is based, in part, on analyses that suggest that 25%–40% of vertically acquired HCV infections clear spontaneously within 4–5 years.

**Methods:**

Data on 179 infants with HCV RNA and/or anti-HCV evidence of vertically acquired infection in 3 prospective European cohorts were investigated. Ages at clearance of infection were estimated taking account of interval censoring and delayed entry. We also investigated clearance in initially HCV RNA–negative infants in whom RNA was not detectable until after 6 weeks.

**Results:**

Clearance rates were initially high then declined slowly. Apparently, many infections clear before they can be confirmed. An estimated 65.9% (95% credible interval [CrI], 50.1–81.6) of confirmed infections cleared by 5 years, at a median 12.4 (CrI, 7.1–18.9) months. If treatment were to begin at age 6 months, 18 months, or 3 years, at least 59.0% (CrI, 42.0–76.9), 39.7% (CrI, 17.9–65.9), and 20.9% (CrI, 4.6–44.8) of those treated would clear without treatment. In 7 (6.6%) confirmed infections, RNA was not detectable until after 6 weeks and not until after 6 months in 2 (1.9%). However, all such cases subsequently cleared.

**Conclusions:**

Most confirmed infection cleared by age 3 years. Treatment before age 3, if it was available, would avoid loss to follow-up but would result in substantial overtreatment.

## Teaser

Based on the largest, purely prospective dataset assembled to date, 66% (50–82) of confirmed vertically acquired hepatitis C virus clears spontaneously by age 5 years, rather than the 25%–40% assumed in guidelines.

The development of highly effective direct-acting antiviral (DAA) treatment for hepatitis C virus (HCV) [1, 2] has raised the possibility that vertically acquired infection, which occurs in 5%–6% of deliveries to HCV RNA–positive monoinfected women [3, 4], could be prevented or treated [5]. Screening of women in pregnancy, followed by treatment in late pregnancy or postnatally, as well as treatment of infected infants, have both been considered [2, 6] and could contribute substantially to the World Health Organization (WHO) target to eliminate HCV by the year 2030 [7]. However, treatment in pregnancy is not currently recommended in spite of good safety profiles [8]. DAA treatment is approved for children aged ≥3 years in the European Union and the United States, and WHO recommendations for lower- and middle-income countries are under review [9–12]. Studies on pediatric formulations and pharmacokinetics are ongoing.

Diagnosis of HCV infection in infants exposed in utero is based largely on the presence of HCV antibodies (anti-HCV) at 18 months [10–13]. Those who are anti-HCV–positive are referred for RNA testing at age 3 years to confirm chronic infection [10].

The policy of deferred testing and treatment is supported by the assumption in recent reviews and guidelines that 25%–40% of vertical infections clear spontaneously by age 5 years [10–12, 14]. However, little attention has been given to the precise timing of clearance during infancy. The literature (see Supplementary Materials for a review) includes studies of infants tested at delivery (Supplementary Table 1) and studies in which children have not been tested regularly from birth and that may miss early clearance Supplementary Tables 2 and 3). In some studies, infants were recruited retrospectively, and inclusion depended on a period of sustained follow-up, potentially resulting in selective inclusion of nonclearers. The 25%–40% clearance estimate derives from the European Pediatric HCV Network (EPHN) study [15], which included 40% retrospectively recruited children.

In addition, previous estimates have not taken account of interval censoring or left truncation in the data. Interval censoring refers to the fact that the age at clearance is never observed, only the ages at the last positive HCV RNA test and the first negative test. Left truncation, also known as “delayed entry,” occurs if the first HCV RNA–positive test is not at or shortly after delivery; this would result in early clearance being missed. Failure to account for either of these features will tend to underestimate clearance and produce a biased picture of its timing.

To better inform guidance on testing and treatment, we sought to obtain unbiased estimates of both the extent and timing of spontaneous clearance of confirmed infection by using a large, purely prospective dataset of infants born to HCV-infected women, followed from birth, using statistical methods appropriate for the interval censored and left-truncated data. We also investigated clearance of viremia, which includes both confirmed infection and cases where evidence for vertically acquired HCV is based on a single positive RNA result. Previous authors [16] have referred to the latter as transient infections.

Finally, only about 40% of vertical infections can be detected at delivery, but most are HCV RNA–positive by 4–8 weeks [17, 18]. However, the literature contains numerous reports of infected infants who remain HCV RNA–negative for several months [19, 20]. This complicates management of infants at risk of vertical infection. Accordingly, we investigated the frequency of late appearance of detectable RNA in infected infants and clearance in this group.

## METHODS

### Sources of Data

We approached the investigators of 21 published prospective studies that followed more than 100 HCV-infected pregnant women and their infants. Three agreed to contribute patient-level data: the EPHN [3, 17, 21]; the British Pediatric Surveillance Unit study, including data from 3 maternity hospitals in Dublin [22]; and the ALHICE study (Alpes-Maritimes, Languedoc, Haute Garonne Infection C chez l’Enfant) [23]. The risk factor distributions, periods of recruitment, and follow-up schedules are summarized in Supplementary Table 4. All infants followed up in these cohorts who met the definition of either confirmed infection or viremia (see below) were included in this analysis. The Faculty of Health Sciences Research Ethics Committee, University of Bristol, approved these analyses of historic data.

### Definitions of Infection and Clearance

#### Confirmed Infection

Confirmed infection was defined as detection of RNA on at least 2 occasions or of anti-HCV after 18 months. Viremia was the same as confirmed infection, except only 1 positive RNA test was required. Confirmed infections are therefore included in viremias. To qualify as a clearer, the child’s last RNA test or, in the absence of an RNA test, the last HCV antibody test had to be negative. In view of the high negative predictive value of these tests [24, 25], only a single marker of clearance was required, either a negative RNA or a negative anti-HCV test. Clearance was considered to have occurred before the date of the first of 2 consecutive negative markers and immediately after the preceding positive RNA test. If there was only 1 (final) marker of clearance, it was considered to have occurred before that marker. Observations were subject to delayed entry; infants were not considered at risk of clearance until after their first positive HCV RNA test.

A 2-marker criterion of clearance was included as a sensitivity analysis in which the single (final) HCV RNA–negative test was considered as a censored observation rather than as a clearance. See the Supplementary Material for further details and supporting information.

### Statistical Methods

Restricted cubic spline time-to-event (“survival”) models [26] were fitted with clearance as the end point, taking interval censoring and delayed entry into account. Cubic splines are flexible models that can fit data smoothly, with the maximum number of “bends” controlled by the user. We allowed for clearance rates with up to 2 turning points (ie, rising initially then falling or vice versa). More complex patterns were considered implausible. Estimation was carried out using Bayesian Markov chain Monte Carlo. Because spline models can be unstable, 3 commonly used parametric models (Weibull, lognormal, and log-logistic) with additional cure parameters were used as sensitivity analyses. Further details of models and program code are shown in the Supplementary Materials.

Based on the estimated clearance curve for confirmed infection, we calculated the proportion of all infected children who would be potentially “overtreated” if treatment was begun at 6, 18, or 36 months in the sense that they would have cleared spontaneously in the absence of treatment (ie, the public health perspective). We also calculated the proportion of treated infections that would have cleared even if treatment began at those ages (ie, the clinical perspective). These calculations assumed that there was no further clearance after age 5 years (see Supplementary Materials).

Finally, we identified infants with at least 1 positive RNA test but whose first RNA test was negative. Those who met the definition of confirmed infection and who were still RNA-negative until after 6 weeks of age were selected for a record review.

## RESULTS

### Age at Testing and Clearance and Numbers at Risk

A total of 106 infants met the definition of having confirmed infection, of whom 36 cleared; 179 were viremic at some point (including those with confirmed infection), of whom 87 cleared. Only 25 (26.4%) of the 106 with confirmed infections and 55 (30.7%) of the 179 with viremia were tested in the first 3 days of life, of whom 9 (36.0%) and 23 (41.8%) were positive, respectively. The median age at the first HCV RNA–positive test was 2.9 months in the confirmed infection cohort and 2.6 months in all those with viremia. Median ages at the first test were 1.0 months and 0.9 months, respectively.

Table 1 documents the ages at which children entered the risk set (the age at the time of their first positive HCV RNA test), alongside the approximate age at clearance, age at loss to follow-up (censoring), and the numbers at risk at each age. Illustrating the delayed entry, the number at risk increases initially as more children are tested and enter the risk set. Note that only 54.8% of those with confirmed infection had entered the risk set by age 3 months and only 57.5% of those with viremia.

**Table 1.** Age at First Test, Age at Clearance, and Number at Risk of Clearance for Confirmed Infection and Viremia

	Confirmed Infection				Viremia			
Age Band	First Tested HCV RNA–Positive (Cumulative % of 106)	Number Cleared^a^ (% of 36)	Number Lost to Follow-up (% of 70)	Number at Risk^b^	First Tested HCV RNA–Positive (cumulative % of 179)	Number Cleared^a^ (% of 87)	Number Lost to Follow-up (% of 92)	Number at Risk^b^
0 d–3 d	9 (8.5)	0 (0)	0 (0)	9	23 (12.8)	0 (0)	4 (4.3)	23
3 d–6 w	26 (33.0)	0 (0)	1 (1.4)	35	45 (38.0)	5 (5.7)	2 (2.2)	64
6 w–3 mo	22 (53.8)	1 (2.8)	0 (0)	56	35 (57.5)	7 (8.0)	5 (5.4)	92
3 mo–6 mo	26 (78.3)	7 (19.4)	10 (14.3)	81	44 (82.1)	28 (23.0)	17 (18.5)	124
6 mo–12 mo	14 (91.5)	7 (19.4)	13 (18.6)	78	22 (94.4)	22 (25.3)	17 (18.5)	101
12 mo–24 mo	6 (97.2)	16 (44.4)	22 (31.4)	64	6 (97.8)	20 (23.0)	21 (22.8)	68
24 mo–36 mo	3 (100)	3 (8.3)	14 (20.0)	29	4 (100)	3 (3.4)	16 (17.4)	31
36 mo–60 mo	0 (100)	2 (5.6)	8 (11.4)	12	0 (100)	2 (2.3)	8 (8.7)	12
>60 mo	0 (100)	0 (0)	2 (2.9)	2	0 (100)	0 (0)	2 (2.2)	2
Total	106	36	70		179	87	92	

Abbreviation: HCV, hepatitis C virus.

The numbers shown cleared no later than in the period indicated but may have cleared in an earlier period, depending on when they first tested positive.

The numbers at risk include those who entered the risk set minus those who cleared or were lost to follow-up in previous time periods.

### Time to Clearance of Infection

**Figure 1.**
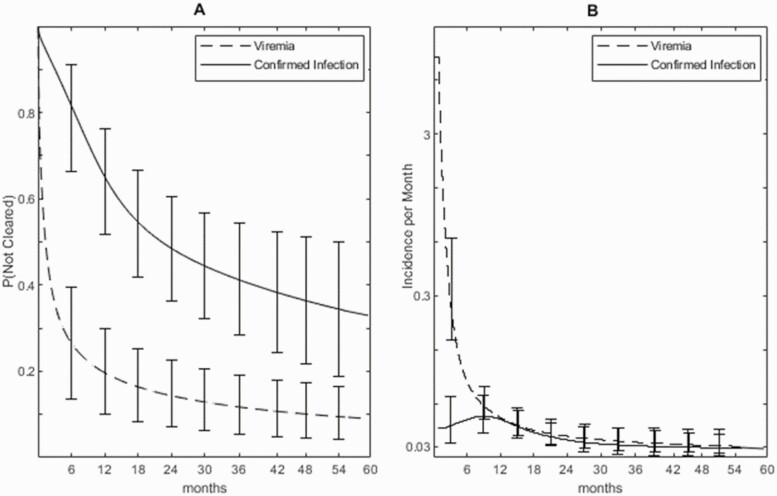
*A,* Proportion of infants remaining infected by age (months) with 95% credible error bars. *B,* Rate of clearance per month with 95% credible error bars on a log scale. Solid line, confirmed infection; dotted line, viremia.

A total of 57.3% (95% credible interval [CrI], 44.7–69.9) of those with confirmed infection cleared by 3 years and 65.9% (CrI, 50.1–81.6) cleared by 5 years (Figure 1A). Among those who cleared in 5 years, the median age at clearance was 12.4 months (CrI, 7.1–18.9). Viremia cleared very rapidly, most within 3 months (Figure 1A), with 79.6% (CrI, 69.0–89.7) clearing by 12 months and 90.6% (CrI, 83.5–95.9) by 5 years. The rate at which clearance occurred declined markedly over time (Figure 1B, note the log scale), although the rate of decline was less with confirmed infection.

### Risk of Overtreatment

Table 2 shows the scenarios with treatment administered at different ages. Taking a public health perspective and assuming no further clearance after age 5, 12.8% (CrI, 3.8–23.1) of all confirmed infections would be overtreated if treatment was begun at 36 months (Table 2) compared with 33.6% at 18 months and 74.3% at 6 months. Taking a clinical perspective, if treatment began at age 36 months, 20.9% (CrI, 4.6–44.8) of remaining infections, that is, of the infections that actually would be treated, would be expected to clear without treatment compared with 39.7% at 18 months and 59.0% at 6 months.

**Table 2.** Projected Proportion “Overtreated” in That They Would be Expected to Clear Without Treatment

Age at Treatment, Mo	Percent of All Infections That Will Clear Without Treatment (95% CrI)	Percent of All Remaining Infections That Will Clear Without Treatment (95% CrI)
6	74.3 (54.3–87.6)	59.0 (42.0–76.9)
18	33.6 (17.0–51.8)	39.7 (17.9–65.9)
36	12.8 (3.8–23.1)	20.9 (4.6–44.8)

Estimated proportion of all confirmed infections that would be potentially overtreated if treatment began at each age (public health perspective). Estimated proportion of remaining confirmed infections overtreated at each age (clinical perspective).

Abbreviation: CrI, credible interval.

### Time to Detectable RNA

A record review of the 7 infants (6.6% of the 106) who were initially HCV RNA–negative and in whom confirmed infection was not detected until after 6 weeks is shown in Table 3. In most cases, there were more than 2 positives and more than 2 final negative tests, so it is unlikely that either the diagnosis of infection or of clearance is due to diagnostic errors. However, in 5 of 7 cases, there was only a single initial negative test over 6 weeks, and there is a possibility these were false-negatives.

**Table 3.** Record Review of 7 Infants Who Met the Definition for Confirmed Infection Who Were Initially RNA-Negative and Remained RNA-Negative Until After 6 Weeks

Case	Age last RNA –ve	No. of initial RNA Negatives > 6 Weeks	Age first RNA Positive	Number of RNA Positives	Age last RNA Positive	Number of final RNA Negatives	Age last antibody Positive	Age first antibody Negative	Ever breastfed	Mode of delivery	Mother HIV	Mother Polymerase Chain Reaction	Infant HIV	Highest ALT, IU/ L	Hepatomegaly
1	2.7	1	6.4	2	9.7	3	9.7	14.1	Yes	EC-S	Neg	Pos	No	40–80	No
2	2.7	1	15.0	2	17.9	2	2.7	23.9	Yes	EC-S	Neg	Neg	No	40–80	No
3	3.0	2	6.0	3	18.0	3	6.0	11.8	Yes	Other	Neg	Pos	No	40–80	No
4	6.1	1	8.8	4	44.0	1	44.0	-	No	EC-S	Neg	NK	No	>80	Yes
5	6.8	2	10.5	3	32.4	3	10.5	14.4	No	Other	Neg	Pos	No	<40	No
**6**	1.9	1	5.8	5	12.3	2	1.9	5.75	No	EC-S	Neg	NK	No	<40	No
**7**	1.7	1	1.9	3	25.9	4	17.9	-	No	Other	Neg	Pos	No	>80	No

Confirmed infection is having at least 2 positive RNA tests. Age is presented in months.

Abbreviations: ALT, alanine transaminase; EC-S, elective cesarean section; HIV, human immunodeficiency virus; NK, not known.

In 4 cases, RNA was not detected until after negative findings at age 3 months and in 2 cases after 6 months. However, all 7 cases eventually became RNA-negative at between 12 and 44 months, 6 meeting the criteria for confirmed clearance. Two, however, were anti-HCV–positive at 17.9 and 44 months, respectively, and had experienced alanine transaminase (ALT) levels >80 IU/L. The risk factors for the 7 infants were unremarkable; 3 of 7 (43%) had breastfed compared with 28% in the whole EPHN cohort (Supplementary Table 4).

### Sensitivity Analyses

For both confirmed infection and viremia, there were no material differences between the preferred cubic spline model and the 3 parametric survival models in model fit, median time to clearance, or estimated age at clearance (Supplementary Tables 5 and 6). Two of the 3 parametric models, the lognormal and log-logistic, allow clearance rates to either rise initially then fall or to fall continuously. For the confirmed infection dataset, both of these parametric models fitted a clearance rate with a brief initial rise, confirming what was seen with the spline model (Figure 1B).

Of the 36 infants who cleared on a 1-marker criterion, 27 cleared on the stricter 2-marker definition. As expected, somewhat less clearance was observed with the 2-marker criterion, but there was little impact on estimated risks of overtreatment if treatment was given at age 36 months.

## DISCUSSION

Based on data from 3 cohort studies following prospectively recruited, vertically infected infants from birth, we estimate that 57.3% (44.7–69.9) of confirmed infection clears by age 3 years and that 65.9% (50.1–81.6) clears by 5 years. These estimates are substantially higher than the 25%–40% clearance rates that are commonly cited [5, 12, 27], which are partly based on datasets in which infants have either not been tested and followed from birth and have therefore missed cases of early clearance or studies that have included retrospectively recruited infants. Studies in which clinics are asked to both retrospectively include children and then follow them prospectively may be especially vulnerable to bias, unless they include all those who have already been discharged or otherwise lost to follow-up at the time the clinic joined the study.

Our finding that 90.6% (95% CrI, 83.5–95.9) of vertically acquired viremia clears by 5 years, mostly before 3 months, is entirely consistent with studies in which children are tested at birth and in which a single RNA test is taken as indicative of potential infection (Supplementary Table 1). In the largest study of this sort [16], 75% had cleared by 1.8 years, and all those who were RNA-positive at delivery, nearly 30% of the total infected, had cleared by 4 months. This illustrates how, by taking delayed entry into account, our analysis is able to recover estimates that are similar to estimates from these studies, even though testing at delivery was infrequent in our dataset.

Current guidance recommends testing for HCV antibodies at 18 months and referral of positives for RNA testing at 3 years to confirm chronic infection prior to treatment [10]. A focus on treatment of chronic infection is reasonable at this time when no treatments are available for those aged <3 years. However, this strategy may not be sustainable in the long term. Loss to follow-up of between 50% and 80% has been reported in a number of US studies [28–30], with less than 50% of children followed to 18 months [31]. Higher follow-up rates should be achievable in well-resourced countries with centralized health systems, but follow-up to even 6 months may be seen as ambitious in lower- and middle-income countries where delayed treatment may, in many cases, result in no treatment with an attendant risk of liver disease and onward transmission [5].

Earlier treatment, were it to become available, could prevent much of the loss to follow-up. However, our results show that this would be at the cost of a substantial degree of overtreatment. Assuming no further clearance after age 5 years, we projected that 33.6% of all confirmed infections and 39.7% of all treated infections would clear spontaneously if treatment was administered at age 18 months and 74.3% and 59.0% at age 6 months. These figures compare with 12.8% and 20.9% overtreatment at the currently recommended age of 3 years. However, if safe, affordable, and effective treatment before age 3 years was available, the additional overtreatment might be considered a price worth paying to avoid risking loss to follow-up and no treatment.

The extent of overtreatment will be greater if further clearance occurs after age 5 years. Clinic-based studies (Supplementary Table 3) suggest 2.3%–29% clearance after 5 years, but these estimates cannot be relied on for reasons given above. More data on rates of clearance after age 3 years would be valuable as the credible intervals on our estimates are wide.

Another reason why delayed treatment is preferred is the difficulty of early diagnosis. While HCV RNA results between 2 and 6 months correlate well with antibody at 18 months [10], it has been reported that 11% (5%–20%) of infected children do not become HCV RNA–positive until after age 3 months [22], and there are isolated reports of late-appearing RNA throughout the literature [19, 20] and even reappearance of infection after apparent clearance [16, 32]. In our study, 7 of 106 confirmed infected infants were still RNA-negative after 6 weeks, including 2 after 6 months. Although it is reassuring that all of these cases eventually cleared, the sample is too small to justify early diagnosis based on HCV RNA. If earlier diagnosis is to be considered, sufficient time must be allowed for all vertical infections to either clear or become manifest.

Given the risk of overtreatment and the difficulties surrounding diagnosis and follow-up, treatment in pregnancy to prevent transmission in pregnancy may be a better strategy than treatment of pediatric infection. However, infants born to mothers treated in pregnancy will still require follow-up and testing, at least until the efficacy of treatment in pregnancy is established.

Although this is the largest purely prospective dataset assembled to date, there are important limitations. Foremost is the historical nature of our dataset, collected between 1994 and 2004 when HCV RNA testing was less accurate and less standardized, so that it was not possible to use quantitative RNA results. This emphasizes the need for additional data. A further difficulty is that confirmed infection is not a well-defined construct; estimates of both the vertical transmission rate and the extent of clearance depend on the precise details of the definition and on the intensity of the follow-up schedule, as noted previously [16, 19]. More frequent testing will result in higher vertical transmission rates and more clearance. However, the frequency of testing after delivery in our data approximates the schedule that might be expected in contemporary trials. The pattern of clearance reported here underscores the need to estimate vertical transmission rates “net” of clearance at different ages. This is the subject of a companion article based on the same 3 cohorts [33].

The significance of a single positive virological marker that clears in the first 3 months is unclear. If these are true infections, as opposed to nonreplicating viral fragments, then the underlying vertical transmission and spontaneous clearance rates are both very much higher than has been thought. Our results and methods may also be relevant to other vertically acquired infections, such as hepatitis B, where there are similar complexities introduced by early clearance of virological markers in the exposed newborn [34, 35].

Here, we have clarified the extent of spontaneous clearance and shed new light on its timing. For the first time, we have set out the statistical and study design considerations required to obtain unbiased estimates and introduced methods for estimating the extent of potential overtreatment. However, our estimates are relatively uncertain, and more accurate information on vertical transmission, clearance rates, and time to detectable RNA is needed. For this, we must await large-scale, randomized trials of early treatment or treatment in pregnancy. In the meantime, routine testing of anti-HCV in antenatal or neonatal samples, now recommended in the United States [36] and shown to be cost-effective at a prevalence of 0.07% [37], would allow HCV RNA–positive infants to be identified and followed until treatment is available.

## Supplementary Data

Supplementary materials are available at *Clinical Infectious Diseases* online. Consisting of data provided by the authors to benefit the reader, the posted materials are not copyedited and are the sole responsibility of the authors, so questions or comments should be addressed to the corresponding author.

ciac255_suppl_Supplementary_Material

## Notes

***Author Contributions.*** A. E. A. conceived of and carried out the analyses with the assistance of F. G. and wrote the first and subsequent drafts of the manuscript. K. S. carried out the literature search and review. A. E. A., A. J., J. C., and D. M. G. were coinvestigators on the HCVAVERT project (Partnership to cure hepatitis C virus (HCV) in mono- and HIV/HCV co-infected pregnant women and prevent VERTical HCV transmission) project, and A. J. was the principal investigator. E. C. was a researcher on the HCVAVERT project. L. P., E. M.-B., D. M. G., and K. B. were senior or principal investigators on the 3 contributing studies: EPHN (European Pediatric HCV Network), ALHICE (Alpes-Maritimes, Languedoc, Haute Garonne Infection C chez l’Enfant), and BPSU (British Pediatric Surveillance Unit). G. I. provided clinical input on hepatology and management of pediatric hepatitis C virus (HCV). Curation of the original data files available for the project was the responsibility of L. P. and C. T. (EPHN); D. M. G., J. C., and K. B. (BPSU); and E. M.-B. (ALHICE). Subsequent data processing was performed by F. G. and A. E. A. All authors critically reviewed and revised drafts as necessary and approved the final version for submission.

***Acknowledgments.*** The authors thank everyone who contributed to the EPHN, BPSU, and ALHICE studies.

***Disclaimer.*** The funding sources did not have any influence on study design, data collection, analysis and interpretation of the data, writing of the manuscript, or the decision to submit for publication.

***Financial support*.** This work was supported by the Medical Research Council (MR/R019746/1) through the Joint Global Health Trials scheme. Work at Great Ormond Street Institute of Child Health was supported by the National Institute of Health Research Biomedical Research Centre, Great Ormond Street Hospital.
