# Supplementary material for: Spontaneous Clearance of Vertically Acquired Hepatitis C Infection: Implications for Testing and Treatment
Source: Clin Infect Dis. 2022 Apr 9;76(5):913–91. doi: 10.1093/cid/ciac255 (PMC9989140; doi:10.1093/cid/ciac255)
Supplement: ciac255_suppl_Supplementary_Material [file ciac255_suppl_supplementary_material.docx]

**SUPPLEMENTARY MATERIALS**

**SPONTANEOUS CLEARANCE OF VERTICALLY ACQUIRED HEPATITIS C INFECTION: IMPLICATIONS FOR TESTING AND TREATMENT**

A E Ades,^1^ Fabiana Gordon,^1^ Karen Scott,^2^ Intira J Collins,^2^ Claire Thorne,^3^ Lucy Pembrey,^4^ Elizabeth Chappell,^2^ Eugènia Mariné-Barjoan,^5^ Karina Butler,^6^ Giuseppe Indolfi,^7^ Diana M Gibb,^2^* Ali Judd,^2^*

^1^ Population Health Sciences, University of Bristol Medical School, Whatley Road Bristol BS8 2PS. ^2^ MRC Clinical Trials Unit, University College London, 90 High Holborn, London WC1V 6LJ. ^3^ Population, Policy and Practice Research and Teaching Department, UCL Great Ormond Street Institute of Child Health, 30 Guilford Street, London WC1N 1EH. *^4^* London School of Hygiene and Tropical Medicine, Keppel Street, London WC1E 7HT. ^5^ Université Côte d'Azur, Public Health Department. Centre Hospitalier Universitaire de Nice, Rte St Antoine de Ginestière BP 3079. 06202 Nice cedex 2. ^6^ Children’s Health Ireland at Crumlin and Temple Street, Dublin, Ireland. ^7^ Meyer Children’s Hospital and Department Neurofarba, University of Florence, Viale Gaetano Pieraccini, 24, 50139 Firenze FI, Italy

*Joint last authors

**LITERATURE REVIEW**

***Literature search***

We searched Pubmed on 19th March 2021 using the search terms: ((hepatitis C[Title/Abstract]) OR (hcv[Title/Abstract])) AND ((natural history[Title/Abstract]) OR (clear*[Title/Abstract]) OR (vertical transmission*[Title/Abstract])) AND ((child*[Title/Abstract]) OR (infant[Title/Abstract]) OR (paediat*[Title/Abstract]) OR (pediat*[Title/Abstract]) OR (mother*[Title/Abstract]) OR (vertical transmission*[Title/Abstract]) OR (pregnan*[Title/Abstract])) NOT (child-pugh[Title/Abstract).

Study reports were included if they clearly described clearance of vertically-acquired HCV in untreated children, with a denominator, number clearing, and method of recruitment. Studies were excluded if the vertical transmission rate was based on less than 20 mothers-child pairs, and or if clearance was based on less than 5 HCV infected infants. Only studies published from 1998 onwards were included, as earlier studies tended to be less clearly described.

The review identified 21 studies (PRISMA flow diagram available on request), which fell into 3 categories (Tables S1, S2, S3). Nine studies reported acquisition and clearance of viremia defined as at least one positive RNA test (Table S1): transmission rates were in the range 12%-70%, and clearance rates 66%-100%. Five of the nine studies also reported results for confirmed infection, with VT rates 3.5%-13% and clearance rates 14%-75%.

There was a second set of 5 studies in which infants were followed from birth but not always tested at delivery (Table S2). Some infections would therefore have cleared without ever being observed. These studies reported clearance rates of 13%-27%, based on analyses that took no account of delayed entry. A third set of seven clinic-based studies followed children from referral: these reported 20%-30% clearance after 3 to 9 years follow-up, but did not account for delayed entry to the risk set (Table S3). These studies would have inevitably underestimated overall clearance as the majority of clearance would have already occurred prior to recruitment into the study. They are also likely to underestimate rates of clearance subsequent to referral and recruitment into the study, for two reasons: firstly, some studies only followed up patients who had not cleared for the initial 6-12 months they were under observation. Secondly, retrospective recruitment of babies for the purpose of further follow-up, even if they have been tested from birth, is likely to result in selective inclusion of children who are still under clinic care at the date when recruitment occurs, and exclusion of those who have already cleared and been discharged.

**Supplementary Table S1.** Infants tested at delivery, VT rates and clearance in Confirmed and Suspected infection combined, and Confirmed infection only.

|  |  | Viraemia | | | Confirmed infection | | | By Age, years |
| --- | --- | --- | --- | --- | --- | --- | --- | --- |
| Lead author (year published) | Number mother-child pairs | VT rate | Number infected infants | **% cleared** | VT rate | Number infected infants | **% cleared** |
| Giacchino (1998)[1] | 45 | 20.0 | 9 | **67** |  |  |  | 1.0 |
| Granovsky (1998)[2] | 122 |  |  |  | 6 | 7 | **14** | 1.5 |
| Ruiz-Extremura (2000)[3] | 67 | 11.9 | 8 | **88** |  |  |  | 1.5 |
| Ketzinal-Gilad (2000)[4] | 23 | 21.7 | 5 | **100** |  |  |  | 0.25 |
| Ceci (2001)[5] | 60 | 50.0 | 30 | **93** | 13.3 | 8 | **75** | 1.0 |
| Mast (2005)[6] | 190 |  |  |  | 4.7 | 9 | **33.3** | 2 |
| Shebl (2009)[7] | 232 | 12.5 | 29 | **72** | 6.5 | 15 | **47** | 5 |
| Ruiz-Extremura (2011)^*[8]^ | 128 | 20.3 | 26 | **65.8** |  |  |  | 6 |
| Bal (2016)[9] | 142 | 66.9 | 95 | **94.7** | 3.5 | 5 | **NR** | 1.5 |

^*^ Positive RNA tests were confirmed within a few days.

**Supplementary Table S2.** Infants followed from birth, but not necessarily tested at birth: delayed entry to risk set not taken into account. These studies reported confirmed infection only.

| Lead author (year published) | Number infected infants | **% cleared** | By Age, years | Comments |
| --- | --- | --- | --- | --- |
| Tovo (2000)[10] | 104 | **13.5** | 4.1 | Inclusion conditional on regular follow-up |
| Resti (2003)[11] | 62 | **19** | 4.8 | Infants recruited between 3 and 12 months, follow-up conditional on infection. |
| Rerksuppaphol (2004)[12] | 16 | **13** | 8.7 | Mean age diagnosis 5 years |
| EPHN 3-broad (2005)[13] | 155 | **24** | 3 | ~40% retrospectively recruited infants enter study aged 2 – 6 months, inclusion conditional on regular follow-up[14] |
| Garazzino (2014)[15] | 45 | **27** | 12 | Inclusion only if anti-HCV at 18 months |

.

**Supplementary Table S3.** Clinic-based studies: follow-up from referral.

| Lead author (year published) | Number of infected infants | Mean age at recruitment, years | Mean length of follow-up, years | Clearance,  % | Comments |
| --- | --- | --- | --- | --- | --- |
| Jara (2003)[16] | 35 | NR | 6.2 | **17.1** | Only included if followed for > 1y |
| Iorio (2005)[17] | 66 | NR | 8.8 | **15** | 39% not vertically infected. Only included if anti-HCV positive for > 6 months |
| EPHN (2005)[13] | 85 | >0.5 | ~3 | **2.3** | Inclusion conditional on regular follow-up[14] |
| Bortolotti (2005)[18] | 270 | 0.25 | NR | **5.6** | 35% not vertically infected. Children with “early occurrence of viraemia clearance” excluded |
| Yeung (2007)[19] | 34 | >2 | 2.8 | **29** | Only included if followed for > 6 months |
| Bortolotti (2008)[20] | 227 | 3.4 | 5 | **11.4** | Only included if followed for > 1y |
| Mizuochi (2018)[21] | 348 | 3.1 | 7.8 | **8.6** | 10% not vertically infected. Only included if followed for > 1y |

**DATA SOURCES**

**Supplementary Table S4.** Period of recruitment, pediatric follow-up schedule, and risk factor distribution in the three cohorts. EPHN European Pediatric HCV Network, BPSU British Pediatric Surveillance Unit, ALHICE Alpes-Maritimes, Languedoc, Haute Garonne Infection C chez l’Enfant. Infants with no laboratory follow-up data were excluded from the table.

|  |  | **EPHN** | | **BPSU** | | **ALHICE** | |
| --- | --- | --- | --- | --- | --- | --- | --- |
| **Period of recruitment** |  | 1999-2004 | | 1994-1999 | | 1999-2003 | |
|  | | | | | | | |
| **Follow-up** |  | Birth, 6w, 3m, 6m, 9m, 12m, 18m, 24, then every 6m | | Birth, 6w, 3m, 6m, 9m, 12m, 18m, 24, then every 6m | | Birth, 3m, 6m, 12m 18m, 24m | |
|  | | | | | | | |
|  |  | **N** | **%** | **N** | **%** | **N** | **%** |
| **Total sample size** |  | 1551 | 100 | 444 | 100 | 214 | 100 |
|  | | | | | | | |
| **Mother HIV** | **+** | 212 | 13.7 | 22 | 5.0 | 55 | 25.7 |
|  | **-** | 1233 | 79.5 | 328 | 73.8 | 157 | 73.3 |
|  | **NK** | 106 | 6.8 | 94 | 21.1 | 2 | 1.0 |
|  | | | | | | | |
| **Mother PCR** | **+** | 469 | 30.2 | 0 | 0 | 138 | 64.5 |
|  | **-** | 184 | 11.9 | 0 | 0 | 62 | 29.0 |
|  | **NK** | 898 | 57.9 | 444 | 100 | 14 | 6.5 |
|  | | | | | | | |
| **Mode of**  **Delivery** | **ECS** | 453 | 29.2 | 27 | 6.1 | 45 | 21.0 |
|  | **Not-ECS** | 1063 | 68.5 | 355 | 80.0 | 169 | 79.0 |
|  | **NK** | 35 | 2.3 | 62 | 14.0 | 0 | 0 |
|  | | | | | | | |
| **Breastfeeding** | **Yes** | 434 | 28 | 31 | 3.8 | 3 | 1.4 |
|  | **No** | 999 | 64.4 | 396 | 89.2 | 173 | 80.8 |
|  | **NK** | 118 | 7.6 | 17 | 7.0 | 38 | 17.8 |

**NK: Not known. ECS: Elective caesarean section**

**DEFINITIONS**

***Infection status***

*Confirmed infection:* Infants were considered infected if they had at least two positive RNA tests (not necessarily consecutive) or anti-HCV positive after 18 months.

*Viremia:* Children with a single positive RNA test were considered to have viraemia, unless they had negative RNA tests when aged > 6weeks, in which case the single positive RNA results were regarded as false positives. Cases of confirmed infection are included within cases of viremia.

***Clearance of infection***

***C****learance:* Children were only considered to have cleared if their last RNA test was negative, or in the absence of any RNA tests their last anti-HCV test was negative. Only a single marker of clearance was required, either a negative RNA test or a negative anti-HCV test. Clearance was considered to have occurred between the date of the first of the two negative markers and the date of the immediately preceding positive RNA test. This was to accommodate cases where a positive RNA follows an initial negative, before a consistent sequence of negative results, and to approximate the clinical requirement for a confirmatory HCV-RNA negative.

For children who had only a single marker of clearance, the date of clearance was between their last negative RNA test and the preceding RNA positive test. The one- and two-marker definitions are designed to allow us to compare results between the two, without changing the definition of when clearance occurs. The only difference between one- and two-marker analyses is that infants with a single, final, HCV-RNA negative test (or a single anti-HCV negative), who are clearers in the main, one-marker, analysis (with clearance occurring between that final negative and the previous positive, are censored observations in the two-marker analysis.

Of the 106 children with confirmed infection, 36 cleared and 27 of these (75%) had two markers of clearance. Of the 179 with viremia, 87 cleared, 81% of who had two markers of clearance

Infants whose first HCV RNA was negative were assumed to clear at the same rate as those who first test was positive, entering the risk set at their first positive test.

**STATISTICAL ANALYSIS OF CLEARANCE**

***Likelihood for interval censored and left truncated data***

We extracted age at first RNA +ve test $a_{i}$, which is the age when individual $i$ enters the risk set; age at last RNA +ve prior to clearance, $l_{i}$; and age at first RNA -ve after clearance,$r_{i}$. If $S(t)$is the survival curve (proportion remaining infected) at age $t$, then the likelihood for an infant $i$who is observed to clear is $L_{i}=\left(S(r_{i})-S(l_{i})\right)/S(a_{i})$. For observations right censored at $l_{i}$ the likelihood is $L_{i}=S(l_{i})/S(a_{i})$.

We fitted natural cubic splines [22]. Knots were placed at the midpoint of the last positive and first negative RNA tests, that is $(l_{i}+r_{i})/2$ [23]. Boundary knots were placed at the smallest and largest midpoints, and two interior knots were placed at the 33^rd^ and 67^th^ centiles of the midpoints. Because spline models can be unstable and dependent on placement of knots, Weibull, lognormal and loglogistic models with a “cure” parameter [24] were fitted as sensitivity analyses, to verify that the cubic spline results were sound. The cure parameter represents the proportion of infected children who (hypothetically) never clear.

***Overtreatment and incidence estimates***

We estimated four measures of “over-treatment”, assuming a 100% effective treatment administered at age $t$

1. Proportion of all infections that would clear spontaneously after $t$months if there was no further clearance after 60 months
2. Proportion of all infections that would clear spontaneously after $t$months if there was 25% additional clearance after 60 months
3. Proportion of all treated infections that would clear spontaneously after $t$months if there was no further clearance after 60 months
4. Proportion of all treated infections that would clear spontaneously after $t$months if there was 25% additional clearance after 60 months

These were calculated respectively as:

$$\begin{matrix} F_{1}(t)=\left(S(t)-S(60)\right)/\left(1-S(60)\right) \\ F_{2}(t)=\left(S(t)-S(60)*0.75\right)/\left(1-S(60)*0.75\right) \\ F_{3}(t)=\left(S(t)-S(60)\right)/S(t) \\ F_{4}(t)=\left(S(t)-S(60)*0.75\right)/S(t) \end{matrix}$$

An approximate mid-period incidence was calculated from weekly estimates of the proportion remaining infected, $S_{w}$:

$$\lambda_{w}=2(S_{w-1}-S_{w})/(S_{w-1}+S_{w})$$

***Estimation***

Estimation was by Bayesian Markov chain Monte Carlo, using WinBUGS 1.4.3 [25]. Cubic spline models converged within 40-60,000 iterations; posterior summaries were based on 400,000 iterations, 80,000 from each of 5 chains, after a burn-in of 80,000.With parametric cure models convergence occurred within 5,000-7,000 iterations, and posterior summaries were based on 120,000 iterations, 40,000 from each of 3 chains, after a “burn-in” of 20,000.

***Goodness of fit***

The posterior mean deviance (minus twice the log likelihood) was used to assess goodness of fit [26].

***WinBUGS code for the cubic spline model***

model{

lambda1<-(kmax-k1)/(kmax-kmin)

lambda2<-(kmax-k2)/(kmax-kmin)

for (i in 1:n[1]) { # loop over all observations

a[i]<-log(A[i])

l[i]<-log(L[i])

##Definition of the splines variables

rcsa1[i]<-a[i]

rcsa2[i]<-pow(max(0,a[i]-k1),3)-lambda1*pow(max(0,a[i]-kmin),3)-(1-lambda1)*pow(max(0,a[i]-kmax),3)

rcsa3[i]<-pow(max(0,a[i]-k2),3)-lambda2*pow(max(0,a[i]-kmin),3)-(1-lambda2)*pow(max(0,a[i]-kmax),3)

rcsl1[i]<-l[i]

rcsl2[i]<-pow(max(0,l[i]-k1),3)-lambda1*pow(max(0,l[i]-kmin),3)-(1-lambda1)*pow(max(0,l[i]-kmax),3)

rcsl3[i]<-pow(max(0,l[i]-k2),3)-lambda2*pow(max(0,l[i]-kmin),3)-(1-lambda2)*pow(max(0,l[i]-kmax),3)

etaa[i]<-gamma[1]+gamma[2]*rcsa1[i]+gamma[3]*rcsa2[i]+gamma[4]*rcsa3[i]

saa[i]<- max(exp(-exp(etaa[i])),sl[i]+.0001)

sa[i] <- equals(A[i],0) + (1-equals(A[i],0)) * saa[i] # sets survival function to 1.0 at t=0

etal[i]<-gamma[1]+gamma[2]*rcsl1[i]+gamma[3]*rcsl2[i]+gamma[4]*rcsl3[i]

sll[i]<-exp(-exp(etal[i]))

sl[i] <- equals(L[i],0) + (1-equals(L[i],0)) * sll[i] # sets survival function to 1.0 at t=0

z[i] <- 0

z[i] ~ dpois(phi[i])

phi[i] <- -log(Lik[i]) + 10000 # WinBUGS "zeros trick"

dv[i] <- 2 * (phi[i]-10000) # deviance

}

for (i in 1:n[3]) { # loop over clearers

r[i]<-log(R[i])

rcsr1[i]<-r[i]

rcsr2[i]<-pow(max(0,r[i]-k1),3)-lambda1*pow(max(0,r[i]-kmin),3)-(1-lambda1)*pow(max(0,r[i]-kmax),3)

rcsr3[i]<-pow(max(0,r[i]-k2),3)-lambda2*pow(max(0,r[i]-kmin),3)-(1-lambda2)*pow(max(0,r[i]-kmax),3)

etar[i]<-gamma[1]+gamma[2]*rcsr1[i]+gamma[3]*rcsr2[i]+gamma[4]*rcsr3[i]

sr[i]<-exp(-exp(etar[i]))

}

for (i in 1:n[3]) { Lik[i] <- max(.0001,(sl[i]-sr[i]) / sa[i]) } # Likelihood clearers

for (i in n[3]+1:n[1]) { Lik[i] <- max(.0001,sl[i] / sa[i] ) } # Likhood non-clearers

sumdev <- sum(dv[])

for (j in 1:4) {gamma[j ] ~ dnorm(0,0.01) } # vague priors

# Computation of Survival function at 3 month intervals

for (t in 1:20) {

x[t]<-log(3*t)

rcs1[t]<-x[t]

rcs2[t]<-pow(max(0,x[t]-k1),3)-lambda1*pow(max(0,x[t]-kmin),3)-(1-lambda1)*pow(max(0,x[t]-kmax),3)

rcs3[t]<-pow(max(0,x[t]-k2),3)-lambda2*pow(max(0,x[t]-kmin),3)-(1-lambda2)*pow(max(0,x[t]-kmax),3)

ETA[t]<- gamma[1]+gamma[2]*rcs1[t]+gamma[3]*rcs2[t]+gamma[4]*rcs3[t]

S[1,t]<-exp(-exp(ETA[t])) # survival function

S[2,t] <- (S[1,t] - S[1,20])/(1-S[1,20]) # Pr clear anyway | ever clears (no more after 5y)

S[3,t] <- (S[1,t] - S[1,20]*0.75)/(1-S[1,20]*0.75) # same 25% more after 5y

S[4,t] <- (S[1,t] - S[1,20]) / S[1,t] # Pr clear anyway | not cleared by t (no more after 5y)

S[5,t] <- (S[1,t] - S[1,20]*0.75) / S[1,t] } # same 25% more after 5y

S[6,1] <- (1 - S[1,1]) * 2 / (S[1,1]+1) # mid-interval incidence

for (t in 2:20) { S[6,t] <- (S[1,t-1] - S[1,t]) * 2 / (S[1,t]+S[1,t-1]) }

# computation of median by interpolation of survival curve at 0.1 month intervals.

for (t in 1:600) {

x2[t] <- log(0.1*t)

rcs12[t]<-x2[t]

rcs22[t]<-pow(max(0,x2[t]-k1),3)-lambda1*pow(max(0,x2[t]-kmin),3)-(1-lambda1)*pow(max(0,x2[t]-kmax),3)

rcs32[t]<-pow(max(0,x2[t]-k2),3)-lambda2*pow(max(0,x2[t]-kmin),3)-(1-lambda2)*pow(max(0,x2[t]-kmax),3)

ETA2[t]<- gamma[1]+gamma[2]*rcs12[t]+gamma[3]*rcs22[t]+gamma[4]*rcs32[t]

S2[t] <- exp(-exp(ETA2[t]))

Z[t] <- (1-S2[t]) / (1-S[1,20]) # median age at clearance | clearance before age 5

J[t] <-t }

md5y <- interp.lin(.5,Z[],J[])

}

#initial values for confirmed infection datasets

list(gamma=c(0,0,0,0))

list(gamma=c(1,-0.1,0,.1))

list(gamma=c(-.2,.2,.1,.1))

list(gamma=c(.5,0,-.1,-.1))

list(gamma=c(0,.2,.1,-.1))

#initial values for viremia datasets

list(gamma=c(1,-0.1,0,.1))

list(gamma=c(-.2,.2,.1,.1))

list(gamma=c(0,.2,.1,-.1))

list(gamma=c(0,-.2,.2,-.1))

list(gamma=c(-.5,.3,0,-.1))

**SENSITIVITY ANALYSIS: RESULTS**

Two sensitivity analyses were carried out on both confirmed infection (Table E1) and viraemia (Table E2): first we looked at sensitivity to model choice (cubic spline or parametric cure models); second, we looked at the alternative definition of clearance based on two markers.

***Confirmed infection***

**Supplementary Table S5.** Sensitivity analysis of clearance of confirmed infection. Posterior mean deviance, median age (months) at clearance among those who clear within 5 years; percent remaining infected at 12, 36 and 60 months; percent (95%CrI) of all infection that clears spontaneously after 36m assuming no further clearance after 60m. * Base-case model

| ***Model*** | ***Posterior mean Deviance*** | ***Median age at Clearance*** | ***Percent Remaining Infected*** | | | ***Percent of all infections that clear spontaneously after 36m, assuming no further clearance after 5 years*** |
| --- | --- | --- | --- | --- | --- | --- |
|  |  |  | ***12m*** | ***36m*** | ***60m*** |  |
| ***Alternative survivals model, one marker of clearance*** | | | | | | |
| **Cubic Spline *** | **228.6** | **12.4 (7.1-18.9)** | **67.4 (53.8-78.3)** | **42.7 (30.2-55.3)** | **34.1 (18.4-49.9)** | **12.8 (3.8-23.1)** |
| Weibull | 227.3 | 10.1 (4.0-16.4) | 63.6 (48.5-75.7) | 41.3 (29.3-53.7) | 34.7 (21.2-49.5) | 9.8 (2.0-19.1) |
| Lognormal | 227.8 | 10.5 (4.2-16.3) | 65.2 (50.5-76.6) | 43.8 (32.1-55.9) | 35.9 (23.8-49.3) | 12.3 (5.9-18.8) |
| Loglogistic | 227.4 | 12.0 (6.5-17.6) | 67.8 (55.3-78.4) | 44.0 (32.6-55.8) | 35.8 (23.6-49.4) | 12.6 (5.8-19.7) |
| ***Two markers of clearance*** | | | | | | |
| Cubic Spline | - | 11.6 (6.3-19.9) | 71.6 (58.7-1.9) | 53.7 (39.8-66.6) | 46.1 (28.1-62.6) | 13.7 (3.6-25.2) |

***Viremia***

**Supplementary Table S6.** Sensitivity analyses of clearance of viremia. Posterior mean deviance, median age (months) at clearance among those who clear within 6 months, and percent clearance. * Base-case model

| ***Model*** | ***Posterior mean Deviance*** | ***Median age at clearance*** | ***Percent remaining not cleared*** | | | | |
| --- | --- | --- | --- | --- | --- | --- | --- |
|  |  |  | ***3m*** | ***6m*** | ***18m*** | ***36m*** | ***60m*** |
| ***Alternative survivals model, one marker of clearance*** | | | | | | | |
| **Cubic Spline** | **383.4** | **1.2 (0.1-2.7)** | **37.1 (19.2-54.7)** | **27.5 (14.2-41.0)** | **17.0 (8.5-26.1)** | **12.2 (5.8-19.8)** | **9.4 (4.1-16.5)** |
| Weibull | 381.7 | 0.6 (0-1.9) | 31.2 (14.4-49.2) | 23.4 (10.6-37.4) | 13.9 (6.4-22.7) | 10.6 (4.7-18.0) | 9.2 (3.8-16.6) |
| Lognormal | 380.9 | 0.7 (0-2.6) | 31.3 (12.9-49.9) | 23.6 (9.7-38.0) | 14.6 (6.1-24.0) | 11.0 (4.5-18.6) | 9.2 (3.6-16.3) |
| Loglogistic | 381.3 | 1.2 (0.2-2.7) | 38.5 (21.6-55.1) | 28.5 (15.9-41.7) | 17.5 (9.7-26.2) | 13.3 (7.2-20.6) | 11.2(5.8-18.2) |
| ***Two markers of clearance*** | | | | | | | |
| Cubic Spline | - | 1.5 (0.1-3.2) | 43.8 (23.7-62.0) | 33.0 (17.8-47.5) | 22.3 (11.8-33.1) | 18.2 (9.2-28.0) | 15.5 (7.4-25.1) |

***References for Supplementary Material***

1. Giacchino R, Tasso L, Timitilli A, et al. Vertical transmission of hepatitis C virus infection: usefulness of viremia detection in HIV-seronegative hepatitis C virus-seropositive mothers. J Pediatr **1998**; 132(1): 167-9.

2. Granovsky MO, Minkoff HL, Tess BH, et al. Hepatitis C virus infection in the mothers and infants cohort study. Pediatrics **1998**; 102(2 Pt 1): 355-9.

3. Ruiz-Extremera A, Salmerón J, Torres C, et al. Follow-up of transmission of hepatitis C to babies of human immunodeficiency virus-negative women: the role of breast-feeding in transmission. Pediatr Infect Dis J **2000**; 19(6): 511-6.

4. Ketzinel-Gilad M, Colodner SL, Hadary R, Granot E, Shouval D, Galun E. Transient transmission of hepatitis C virus from mothers to newborns. Eur J Clin Microbiol Infect Dis **2000**; 19(4): 267-74.

5. Ceci O, Margiotta M, Marello F, et al. Vertical transmission of hepatitis C virus in a cohort of 2,447 HIV-seronegative pregnant women: a 24-month prospective study. J Pediatr Gastroenterol Nutr **2001**; 33(5): 570-5.

6. Mast EE, Hwang LY, Seto DS, et al. Risk factors for perinatal transmission of hepatitis C virus (HCV) and the natural history of HCV infection acquired in infancy. J Infect Dis **2005**; 192(11): 1880-9.

7. Shebl FM, El-Kamary SS, Saleh DA, et al. Prospective cohort study of mother-to-infant infection and clearance of hepatitis C in rural Egyptian villages. J Med Virol **2009**; 81(6): 1024-31.

8. Ruiz-Extremera A, Muñoz-Gámez JA, Salmerón-Ruiz MA, et al. Genetic variation in interleukin 28B with respect to vertical transmission of hepatitis C virus and spontaneous clearance in HCV-infected children. Hepatology **2011**; 53(6): 1830-8.

9. Bal A, Petrova A. Single Clinical Practice's Report of Testing Initiation, Antibody Clearance, and Transmission of Hepatitis C Virus (HCV) in Infants of Chronically HCV-Infected Mothers. Open Forum Infect Dis **2016**; 3(1): ofw021.

10. Tovo PA, Pembrey LJ, Newell ML. Persistence rate and progression of vertically acquired hepatitis C infection. European Paediatric Hepatitis C Virus Infection. J Infect Dis **2000**; 181(2): 419-24.

11. Resti M, Jara P, Hierro L, et al. Clinical features and progression of perinatally acquired hepatitis C virus infection. J Med Virol **2003**; 70(3): 373-7.

12. Rerksuppaphol S, Hardikar W, Dore GJ. Long-term outcome of vertically acquired and post-transfusion hepatitis C infection in children. J Gastroenterol Hepatol **2004**; 19(12): 1357-62.

13. European Paediatric Hepatitis C Virus Network. Three broad modalities in the natural history of vertically acquired hepatitis C virus infection. Clin Infect Dis **2005**; 41(1): 45-51.

14. Pembrey LJ, University of L. Mother-to-child transmission of hepatitis C virus : a European epidemiological collaboration. London: University of London, **2006**.

15. Garazzino S, Calitri C, Versace A, et al. Natural history of vertically acquired HCV infection and associated autoimmune phenomena. Eur J Pediatr **2014**; 173(8): 1025-31.

16. Jara P, Resti M, Hierro L, et al. Chronic hepatitis C virus infection in childhood: clinical patterns and evolution in 224 white children. Clin Infect Dis **2003**; 36(3): 275-80.

17. Iorio R, Giannattasio A, Sepe A, Terracciano LM, Vecchione R, Vegnente A. Chronic hepatitis C in childhood: an 18-year experience. Clin Infect Dis **2005**; 41(10): 1431-7.

18. Bortolotti F, Resti M, Marcellini M, et al. Hepatitis C virus (HCV) genotypes in 373 Italian children with HCV infection: changing distribution and correlation with clinical features and outcome. Gut **2005**; 54(6): 852-7.

19. Yeung LT, To T, King SM, Roberts EA. Spontaneous clearance of childhood hepatitis C virus infection. J Viral Hepat **2007**; 14(11): 797-805.

20. Bortolotti F, Verucchi G, Cammà C, et al. Long-term course of chronic hepatitis C in children: from viral clearance to end-stage liver disease. Gastroenterology **2008**; 134(7): 1900-7.

21. Mizuochi T, Takano T, Yanagi T, et al. Epidemiologic features of 348 children with hepatitis C virus infection over a 30-year period: a nationwide survey in Japan. J Gastroenterol **2018**; 53(3): 419-26.

22. Royston P, Parmar MK. Flexible parametric proportional-hazards and proportional-odds models for censored survival data, with application to prognostic modelling and estimation of treatment effects. Stat Med **2002**; 21(15): 2175-97.

23. Johansen MN, Lundbye-Christensen S, Larsen JM, Parner ET. Regression models for interval censored data using parametric pseudo-observations. BMC Med Res Methodol **2021**; 21(1): 36.

24. Lambert PC, Thompson JR, Weston CL, Dickman PW. Estimating and modeling the cure fraction in population-based cancer survival analysis. Biostatistics **2007**; 8(3): 576-94.

25. Lunn DJ, Thomas A, Best N, Spiegelhalter D. WinBUGS - A Bayesian modelling framework: Concepts, structure, and extensibility. Statistics and Computing **2000**; 10(4): 325-37.

26. Spiegelhalter DJ, Best NG, Carlin BP, Van Der Linde A. Bayesian measures of model complexity and fit. Journal of the Royal Statistical Society: Series B (Statistical Methodology) **2002**; 64(4): 583-639.
